# Plasma Diaphanous Related Formin 1 Levels Are Associated with Altered Glucose Metabolism and Insulin Resistance in Patients with Polycystic Ovary Syndrome: A Case Control Study

**DOI:** 10.1155/2022/9620423

**Published:** 2022-02-11

**Authors:** Xing Li, Mingyu Liao, Jiaqing Shao, Weixin Li, Liu Shi, Dong Wang, Juan Ni, Qiuyue Shen, Fan Yang, Guiliang Peng, Ling Zhou, Yuling Zhang, Zheng Sun, Hongting Zheng, Min Long

**Affiliations:** ^1^Department of Endocrinology, Jinling Hospital, Medical School of Nanjing University, Nanjing, China; ^2^Department of Endocrinology, Jinling Hospital, Nanjing Medical University, Nanjing, China; ^3^Department of Endocrinology, Translational Research Key Laboratory for Diabetes, Xinqiao Hospital, Army Medical University, Chongqing, China; ^4^Department of Pulmonary and Critical Care Medicine, Jinling Hospital, Medical School of Nanjing University, Nanjing, China; ^5^National Clinical Research Center of Kidney Diseases, Jinling Hospital, Medical School of Nanjing University, Nanjing, China; ^6^Department of Medicine, Division of Diabetes, Endocrinology and Metabolism, Baylor College of Medicine, Houston, Texas, USA; ^7^Department of Molecular and Cellular Biology, Baylor College of Medicine, Houston, Texas, USA

## Abstract

**Background:**

Diaphanous related formin 1 (DIAPH1) is a novel component of advanced glycation end product (AGE) signal transduction that was recently found to participate in diabetes-related disorders, obesity, and androgen hormones. We investigated whether plasma DIAPH1 levels were a potential prognostic predictor for polycystic ovary syndrome (PCOS).

**Methods:**

The levels of circulating plasma DIAPH1 and indicators of glucose, insulin, lipid metabolism, liver enzymes, kidney function, sex hormones, and inflammation were measured in 75 patients with PCOS and 77 healthy participants. All of the participants were divided into normal-weight (NW) and overweight/obese (OW) subgroups. Statistical analyses were performed with R studio.

**Results:**

PCOS patients manifested hyperandrogenism, increased luteinizing hormone/follicle-stimulating hormone (LH/FSH), and accumulated body fat and insulin resistance. Plasma DIAPH1 levels were significantly decreased in women with PCOS compared to control participants, and DIAPH1 levels were distinctly reduced in OW PCOS compared to OW control subjects (*P* < 0.001). DIAPH1 levels correlated with fasting blood glucose (FBG), total cholesterol (TC), the homeostasis model assessment of *β*-cell function (HOMA-*β*), and LH/FSH in all participants (FBG: *r* = 0.351, *P* < 0.0001; TC: *r* = 0.178, *P* = 0.029; HOMA-*β*: *r* = −0.211, *P* = 0.009; LH/FSH: *r* = −0.172, *P* = 0.040). Multivariate logistic regression analysis revealed that plasma DIAPH1 levels were an independent risk factor for PCOS. A model containing DIAPH1, BMI, FBG, and testosterone was constructed to predict the risk of PCOS, with a sensitivity of 92.0% and a specificity of 80.9%. A nomogram was constructed to facilitate clinical diagnosis.

**Conclusions:**

These findings suggest the association of plasma DIAPH1 with glucose metabolism, insulin resistance, and sex hormones and support DIAPH1 as a potential predictive factor for PCOS.

## 1. Introduction

Polycystic ovary syndrome (PCOS) is a metabolic and reproductive disorder that is characterized by ovulation dysfunction, hyperandrogenism, and polycystic ovary changes, which are commonly accompanied by insulin resistance (IR) and compensatory hyperinsulinemia [1]. The clinical manifestations are generally irregular menstruation or amenorrhea, obesity, hirsute, and acne [1]. PCOS patients are at risk of metabolic disorders, such as diabetes, hypertension, and other cardiovascular and cerebrovascular complications [2–4]. Women with PCOS have systemic chronic inflammatory conditions, which are closely related to IR, hyperandrogenism, and obesity [5]. Several novel circulating indicators reveal the inflammatory state of the body [6]. Recent studies revealed that elevated levels of advanced glycation end products (AGEs), a group of glycated proteins or lipids after exposure to sugars, represented the inflammatory state in PCOS, which could be aggravated by obesity and hyperinsulinemia [7, 8]. The role of novel markers of AGE signaling in the pathology and development of PCOS are not clear.

Diaphanous related formin 1 (DIAPH1) is a recently identified component of the AGE signaling pathway that is basically a member of the formin family of actin-polymerizing proteins and effector protein of the small GTPase RhoA [9]. DIAPH1 was previously implicated in actin remodeling during the migration of immune cells [10]. There are a series of DIAPH1-related diseases due to DIAPH1 variants or deficiency, such as microcephaly syndrome (SCBMS), immunodeficiency, mitochondrial dysfunction [11], macrothrombocytopenia, and hearing loss [12]. Recent studies reported that DIAPH1 was involved in glucose metabolism because the FH1 (formin homology 1) domain of DIAPH1 bound to the receptor for advanced glycation end products (RAGE) and was required for RAGE signal transduction [13, 14]. RAGE is a multiligand cell surface macromolecule and a signal transduction receptor that senses AGEs and plays a central role in the etiology of diabetes complications, inflammation, neurodegeneration, and PCOS [15, 16].

Deletion of DIAPH1 protected against structural and functional abnormalities in the murine diabetic kidney [17] and cardiac ischemia–reperfusion injury [14]. DIAPH1 is also required for steroid hormone biosynthesis and the secretion of adrenal androgens [18, 19]. However, investigations of DIAPH1 in association with PCOS are rare. Because DIAPH1 participates in the AGE signaling that aggravates inflammation and IR in PCOS and hormone reproduction, we examined the potential connection of DIAPH1 with PCOS.

To elucidate the function of DIAPH1 in PCOS, the present cross-sectional study evaluated the plasma DIAPH1 concentrations in PCOS patients, normal-weight, and overweight/obese subgroups. The associations of DIAPH1 with metabolic parameters, sex hormones, and inflammatory markers were detected. The study also evaluated whether DIAPH1 was a reference marker for susceptible people and whether these clinical indicators in combination with DIAPH1 may be used as diagnostic factors for predicting the risk of PCOS.

## 2. Materials and Methods

### 2.1. Participants

The clinical trial started from April 2017 to October 2020. This study was authorized by the Human Research Ethics Committee of Xinqiao Hospital (no. 2020-124-01) and registered in Chinese Clinical Trial Registry (no. ChiCTR-ROC-17010719). Informed consent was procured from all subjects. 75 women with PCOS (19 normal-weight and 56 overweight/obese) and 77 healthy control subjects (47 normal-weight and 30 overweight/obese) were recruited. Overweight/obese was defined as BMI ≥ 24 kg/m^2^ in this study since all subjects were of Asian ethnicity [20]. The participants were categorized into subgroups as normal-weight (NW: BMI < 24 kg/m^2^) and overweight/obese (OW: BMI ≥ 24 kg/m^2^).

### 2.2. Inclusion and Exclusion Criteria

PCOS was diagnosed according to the Rotterdam criteria [21] with the presence of at least two of the following three features: oligo- and/or anovulation, hyperandrogenism (elevated testosterone or dehydroepiandrosterone sulfate, severe acne, androgenic alopecia, or clinical hirsutism), and polycystic ovaries (ovarian volume > 10 mL and/or at least 12 small follicles with diameter between 2 mm to 9 mm in at least one ovary). According to the standard for diagnosis formulated by the Ministry of Health based on characteristics of PCOS patients in China, irregular uterine bleeding, irregular menstruation, or amenorrhea are also the necessary conditions for the diagnosis of PCOS, after excluding other diseases that may cause hyperandrogenism and ovulation disorders, such as nonclassic hyperprolactinemia, congenital adrenal hyperplasia, androgen-secreting tumors, 21-hydroxylase deficiency (21-OHD), Cushing syndrome, and thyroid disease [22]. The other exclusion criteria were smoking, alcohol intake greater than 20 g/day, type 1 diabetes, malignant diseases, heart failure, active liver disease, hepatic failure, renal failure, and other reproductive pathologies, such as a history of recurrent abnormal intrauterine cavity, spontaneous abortion, or unilateral oophorectomy. The control participants were recruited from healthy women who underwent a physical examination displaying normal menstrual cycle and clinical parameters. The pelvic examination and ultrasonography results were normal. The participants were excluded at the age below 14 years or over 50 years. None of them was taking medications (such as antiobesity drugs, antidiabetic drugs, insulin sensitizers, oral contraceptives, glucocorticoids, and ovulation induction agents) that could affect metabolic parameters or sex hormones for at least half a year before recruiting.

### 2.3. Data Collection

Clinical data, including height, weight, waist circumference, hip circumference, systolic blood pressure (SBP), and diastolic blood pressure (DBP) were collected. The body mass index (BMI) was calculated as weight in kilograms divided by height in meters square (kg/m^2^). Blood samples were obtained at the follicular phase of the menstrual period, after an overnight fast. Glucose and insulin metabolic profiles including fasting blood glucose (FBG), fasting insulin (FINS), and glycosylated hemoglobin A1c (HbA1c) were measured. The homeostasis model assessment of IR (HOMA-IR) and insulin secretion (HOMA-*β*) was calculated using the following equations [23]: HOMA − IR = FINS (mU/mL) × FBG (mmol/L)/22.5 and HOMA − *β* (%) = 20 × FINS/(FBG–3.5). An oral glucose tolerance test (OGTT) and insulin release test were conducted for PCOS subjects. Each participant consumed 75-g glucose beverage in 5 min, blood samples were collected before the start of the test (0 min) and at 30, 60, 90, 120, and 180 minutes after the 75 g glucose intake. The insulin level was measured later from plasma stored at –80°C. The insulin area under the curve (AUC-Insulin) was calculated. Lipid profiles including total cholesterol (TC), triglyceride (TG), high-density lipoprotein cholesterol (HDL-C), and low-density lipoprotein cholesterol (LDL-C) were measured. Liver enzymes and indicators of kidney function were detected, such as glutamic pyruvic transaminase (ALT), glutamic oxaloacetic transaminase (AST), *γ*-glutamyl transferase (*γ*-GGT), uric acid (UA), creatinine, and blood urea nitrogen (BUN). Sex hormone profiles such as luteinizing hormone (LH), follicle-stimulating hormone (FSH), testosterone, estradiol, progesterone, prolactin, and inflammatory marker WBC were measured in all participants. The sex hormone-binding globulin (SHBG), dehydroepiandrosterone sulfate (DHEA-S), and other inflammatory markers such as C-reactive protein (CRP), tumor necrosis factor- (TNF-) *α*, interleukin- (IL-) 6, and IL-8 were measured in PCOS participants. The free androgen index (FAI) was calculated using the following equation: FAI = Total Testosterone (nmol/L) x 100/SHBG (nmol/L). All the test method and equipment of clinical indicators were displayed in Supplementary Table 7.

### 2.4. Plasma Collection and DIAPH1 Measurement

The whole blood was collected using EDTA anticoagulant tube, and the plasma separation was performed within 1 h: centrifugation at 3000 RPM for 10 min at room temperature, the upper layer plasma was divided in the RNase-free and DNase-free centrifuge tubes, then, freezed at -80°C immediately. Plasma DIAPH1 was determined by the ELISA kit (catalog no: SEJ265Hu; Cloud-clone, Wuhan, China). The kit had a sensitivity of 0.055 ng/mL, with a range between 0.156 ng/mL and 10 ng/mL. The intra-assay and interassay variations were 10% and 12%, respectively.

### 2.5. Statistical Analysis

Analyses were conducted by R studio version 1.3.1093. Normally distributed continuous variables are described as mean ± standard deviation, and median with the interquartile range (25–75%) is for nonnormally distributed continuous variables. For variables with a normal distribution, an independent samples *t*-test was performed to compare variables between two groups; a one-way ANOVA followed by Tukey multiple comparison test was performed among the four subgroups. For nonnormally distributed continuous variables, the Mann–Whitney *U* test was performed to compare variables between the two groups; the Kruskal-Wallis test followed by pairwise comparisons using BWS all-pairs test was performed for the four subgroups. The relationships between variables were analyzed using Spearman correlation analysis. Diagnostic values were assessed using sensitivities, specificities, and the areas under the receiver operating characteristic curves (AUC-ROC). The univariate and multivariate logistic regression were performed to assess the association of the variables with diagnosis. A nomogram was derived using the predictors from the multivariate analysis for relating the risk of having PCOS to decreasing concentrations of plasma DIAPH1. *P* value < 0.05 was defined as statistically significant.

## 3. Results

### 3.1. Clinical Parameters of the PCOS and the Control Groups

As shown in Table 1, the age of participants in the PCOS group (26 years old) was younger than the healthy group because PCOS exists in women of reproductive age. The BMI, waist circumference, and waist-to-hip ratio (WHR) were significantly higher in women with PCOS than women without PCOS (*P* < 0.001). The levels of SBP and DBP were slightly higher in women with PCOS (*P* < 0.05 or *P* < 0.001). Glucose and insulin metabolic parameters, including FINS, HbA1c, HOMA-IR, and HOMA-*β*, were all significantly higher (94.74%, 0.78%, 100.61%, and 65.31%, respectively) in women with PCOS (*P* < 0.001), which revealed that PCOS patients had obvious insulin resistance (IR) and compensatory hyperinsulinemia. Women with PCOS had significantly elevated TG and LDL-C and lower HDL-C compared to women in the control group (*P* < 0.001 or *P* < 0.01). PCOS participants showed increased liver enzymes ALT, AST, and *γ*-GGT and renal function indices, such as UA and creatinine (*P* < 0.05 or *P* < 0.001). The WBC level was higher in the PCOS group (*P* < 0.001), which revealed chronic inflammation in PCOS patients. PCOS patients had excess androgen levels with increased LH and attenuated periodic female hormone secretion, such as significantly higher levels of testosterone, LH and LH/FSH (2.34-, 1.81-, and 1.91-fold compared to the control, respectively, *P* < 0.001), and lower levels of estradiol and progesterone (65.0% and 30.08% lower, respectively, *P* < 0.001) (Table 1).

The incidence of overweight and obesity appears more often in PCOS patients than control groups [24]. Therefore, we stratified participants into normal-weight (NW) and overweight/obese (OW) subgroups based on BMI to observe specific parameters. Participants with BMI ≥ 24 kg/m^2^ were considered OW [20]. The participants in each subgroup were age-matched. As the level of BMI increased, waist circumference and WHR levels increased with the level of BMI in the OW subgroups compared to the NW subgroups. The levels of glucose and insulin indicators (FBG, FINS, HbA1c, and HOMA-IR) also increased significantly in the control and PCOS groups (a *P* < 0.05 for NW-Con vs. OW-Con; c *P* < 0.05 for NW-PCOS vs. OW-PCOS, Table 1). For all NW participants, women with PCOS exhibited a risk of abdominal obesity and metabolic disorder compared to women without PCOS because they had greater waist circumference, higher levels of FINS, HbA1c, HOMA-IR, UA, creatinine, and sex hormone dysregulation (b *P* < 0.05 for NW-Con vs. NW-PCOS, Table 1). Notably, metabolic disorders accelerated hyperandrogenism in OW participants because the levels of metabolic indicators (FINS, HbA1c, HOMA-IR, HOMA-*β*, TG, liver enzymes, and UA), testosterone, LH, LH/FSH, and WBC were all robustly elevated in women with PCOS compared to women without PCOS (d *P* < 0.05 for OW-Con vs. OW-PCOS, Table 1). Women in the OW-PCOS subgroup exhibited significantly attenuated insulin sensitivity and enhanced androgen production, such as increased 60 min and 120 min insulin levels of the OGTT (66.24% and 2.01-fold, respectively), decreased SHBG, and elevated FAI, compared to women in the NW-PCOS subgroup (*P* < 0.001 or *P* < 0.01, Supplementary Table 1).

## 4. The Plasma DIAPH1 Level and Its Associations with Metabolic Profiles and Sex Hormones

**Figure 1:**
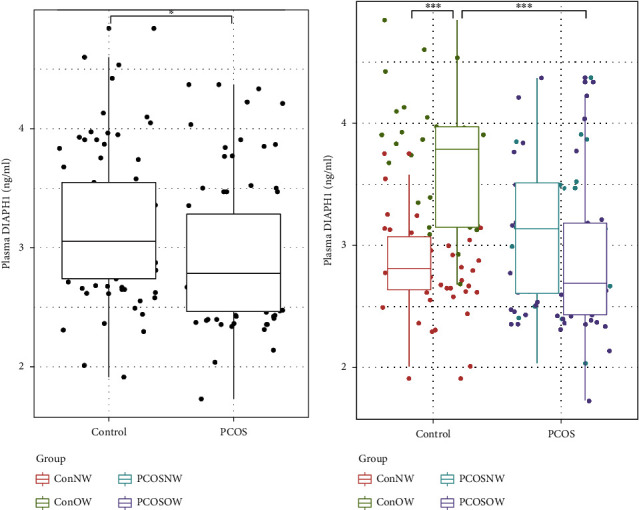
Plasma DIAPH1 levels in PCOS and healthy subjects. (a) The DIAPH1 levels in healthy controls and PCOS subjects, control *n* = 77, PCOS *n* = 75. (b) DIAPH1 levels in subjects divided into normal-weight and overweight subgroups, Con-NW *n* = 47; Con-OW *n* = 19; PCOS-NW *n* = 30; PCOS-OW *n* = 56. Data are presented as interquartile ranges (25–75%). The Mann–Whitney *U* test was performed to compare variables between the two groups. The Kruskal-Wallis test followed by pairwise comparisons using the BWS all-pairs test was performed for the four subgroups. ^∗^*P* < 0.05, ^∗∗^*P* < 0.01, ^∗∗∗^*P* < 0.001.

The level of plasma DIAPH1 was significantly reduced in the PCOS group compared to the control group (*P* < 0.05; Figure 1(a)). OW-Con participants showed significantly elevated DIAPH1 compared to NW-Con participants (1.35-fold, *P* < 0.001). The DIAPH1 levels decreased 27.44% in OW-PCOS patients compared to OW-Con participants (*P* < 0.001). Among all participants, the plasma DIAPH1 levels positively correlated with the levels of WHR, FBG, and TC and negatively correlated with HOMA-*β* and LH/FSH (*P* < 0.05 or *P* < 0.01 or *P* < 0.001, Table 2). We defined the overweight participants (with or without PCOS) as the OW group. Positive correlations of DIAPH1 with FBG and inverse correlations of DIAPH1 with HOMA-*β* also existed in the OW group and PCOS group (*P* < 0.05 or *P* < 0.01 or *P* < 0.001, Table 2). Plasma DIAPH1 levels in OW women were associated with the levels of UA and sex hormone indicators, such as testosterone, E2, P, and LH/FSH (*P* < 0.05 or *P* < 0.01, Table 2). The correlations between DIAPH1 and indicators (FBG, HbA1c, HOMA-*β*, LH/FSH, *γ*-GGT, and TNF-*α*) in PCOS patients are also shown in Supplementary Figure 1, Table 2, and Supplementary Table 2. Among these indicators, FBG and HOMA-*β* exhibited larger coefficients and significance: FBG levels positively correlated with DIAPH1 levels (*r* = 0.46, *P* < 0.0001), and HOMA-*β* levels inversely correlated with DIAPH1 levels (*r* = −0.33, *P* < 0.01) (Supplementary Figure 1).

## 5. The Connection between DIAPH1 and FBG or HOMA-*β* in Women with PCOS

**Figure 2:**
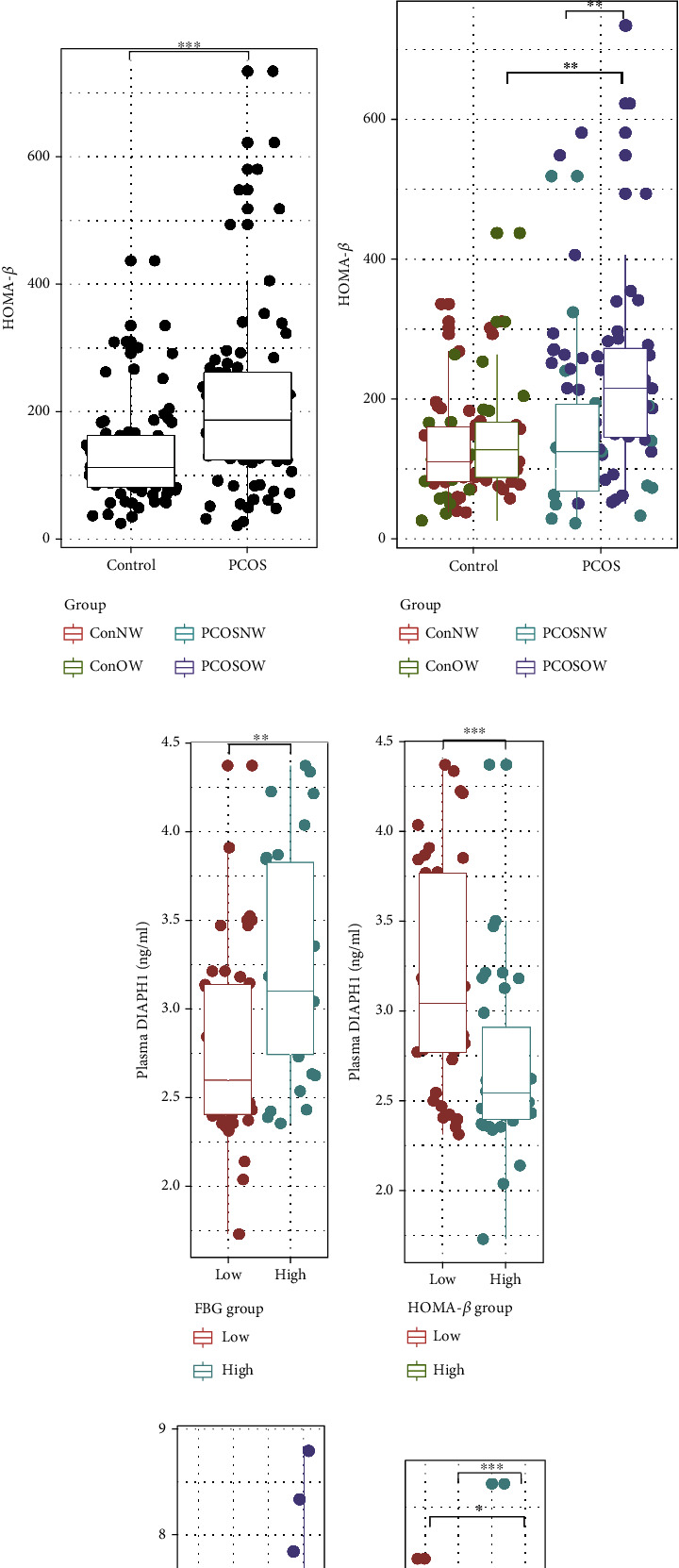
FBG and HOMA-*β* levels in the PCOS and control groups and DIAPH1 quantile groups. (a) FBG levels in the control and PCOS group. (b) FBG levels in the normal-weight (NW) and overweight/obese (OW) subgroups of the control and PCOS groups. (c) HOMA-*β* levels in the control and PCOS group. (d) HOMA-*β* levels in the normal-weight (NW) and overweight/obese (OW) subgroups of the control and PCOS groups. (e) DIAPH1 levels by FBG in the PCOS group: FBG < 5 represented a relatively low level of FBG, and FBG > 5 represented a relatively high level of FBG. (f) DIAPH1 levels by HOMA-*β* in the PCOS group: HOMA − *β* < 200 represented a relatively low level of HOMA-*β*, and HOMA − *β* > 200 represented a relatively high level of HOMA-*β*. (g) FBG levels by DIAPH1 quartiles in the PCOS group. (h) HOMA-*β* levels by DIAPH1 quartiles in the PCOS group. Data are presented as interquartile ranges (25–75%). The Mann–Whitney *U* test was performed to compare variables between the two groups. The Kruskal-Wallis test followed by pairwise comparisons using the BWS all-pairs test was performed for the four subgroups. ^∗^*P* < 0.05, ^∗∗^*P* < 0.01, ^∗∗∗^*P* < 0.001.

The levels of FBG increased significantly in the OW subgroups compared to the NW subgroups (Figures 2(a) and 2(b)). The levels of HOMA-*β* were significantly increased in PCOS and OW-PCOS participants (Figures 2(c) and 2(d)). FBG and HOMA-*β* are closely related to DIAPH1, and glucose intolerance and IR are important characteristics of PCOS patients. Therefore, we examined whether the FBG or HOMA-*β* level affected the DIAPH1 level. First, we compared the DIAPH1 levels in FBG- or HOMA-*β*-divided subgroups in PCOS patients to identify whether DIAPH1 levels changed with FBG or HOMA-*β* (Figures 2(e) and 2(f)). The DIAPH1 levels were higher in women with FBG > 5 compared to women with FBG < 5 (3.10 ng/mL vs. 2.60 ng/mL *P* < 0.01, Figure 2(e)). DIAPH1 levels were lower in participants with HOMA − *β* > 200 compared to participants with HOMA − *β* < 200 (2.54 ng/mL vs. 3.04 ng/mL, *P* < 0.001, Figure 2(f)). The baseline data according to FBG or HOMA-*β* in women with PCOS are shown in Supplementary Tables 3 and 4. To examine the effects of DIAPH1 in women with PCOS, we further divided the subjects into DIAPH1-quantile subgroups according to DIAPH1 levels (Supplementary Table 5) and compared the FBG and HOMA-*β* levels (Figures 2(g) and 2(h)). FBG levels in women with PCOS were not significantly different between the DIAPH1-quantile subgroups (Figure 2(g)), but HOMA-*β* levels were significantly higher in PCOS women with lower DIAPH1 levels in the first (2.25-fold, *P* < 0.05 for Q1 vs. Q4) and second quantiles (2.28-fold, *P* < 0.05 for Q2 vs. Q4) compared to women with the highest DIAPH1 levels in the fourth quartile (Figure 2(h)). These results are consistent with the previous result that HOMA-*β* negatively correlated with DIAPH1. Notably, the TC levels differently changed in the DIAPH1 quantile subgroups (*P* < 0.05 for Q1 vs. Q3). The baseline data of the DIAPH1 quantile subgroups in PCOS women are shown in Supplementary Table 5.

## 6. Prognostic Model for PCOS

**Figure 3:**
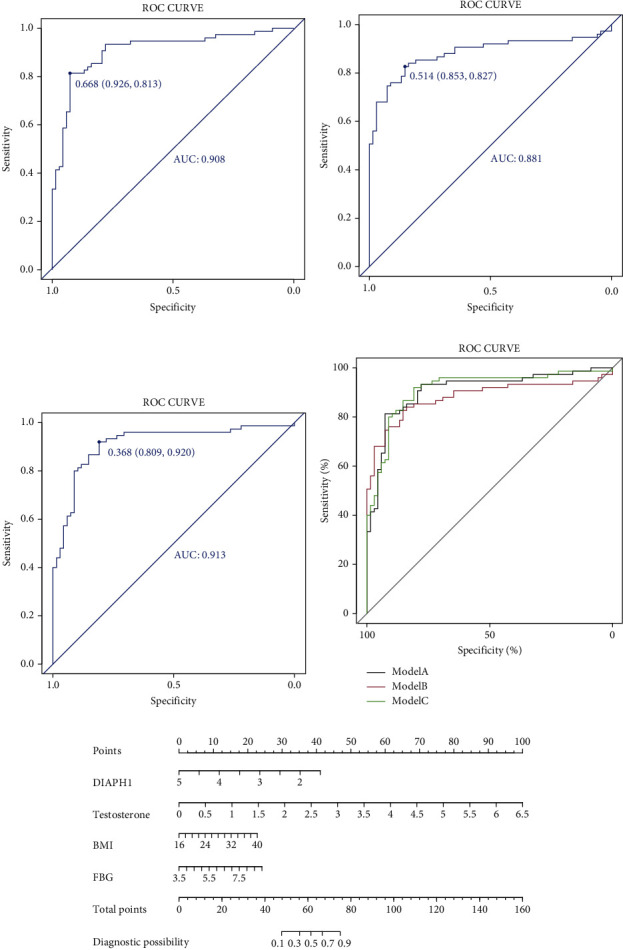
The diagnosis of PCOS using clinical indicators and plasma DIAPH1. (a)–(c) The ROC curves in 3 different models. (a) Model A: BMI + HOMA − *β* + testosterone + DIAPH1; (b) model B: BMI + FBG + LH/FSH + DIAPH1; (c): model C: BMI + FBG + testosterone + DIAPH1. (d) The combination of ROC curves in 3 models. (e) The logistic regression-based nomogram is based on variables in model C. Instructions: according to patient values at each axis, vertical lines could be drawn to the point axis to determine the score/point for each variable. The vertical lines of the calculated total points line referred to the diagnostic possibility of PCOS. Example: during the child-bearing period, the woman had a BMI of 26 kg/m2 and a plasma DIAPH1 level of 2.5 mg/mL, FBG level of 7 mmol/L, and testosterone level of 1.0 nmol/L; the points for the DIAPH1 points were 30, BMI was approximately 10, FBG points were 15, and testosterone points were 23. The total points are summed as 88, and the possibility of having PCOS is approximately 88%. ROC curve: receiver operating characteristic curve.

Plasma DIAPH1 was significantly associated with various parameters of clinical importance in this study. For the analysis of PCOS risk, several indicators were chosen for the construction of regression models. Model A contained BMI, HOMA-*β*, testosterone, and DIAPH1. Model B contained BMI, FBG, LH/FSH, and DIAPH1, and model C contained BMI, FBG, testosterone, and DIAPH1 (Supplementary Table 6). Receiver operating characteristic (ROC) curves combining different models were derived from regression analysis for the diagnosis of PCOS (Figures 3(a)–3(c)). Among the three models, the area under the ROC curve of model C was the highest at 0.913, and the odds ratio (OR) was 0.21. The 95% confidence interval was between 0.08 and 0.49, with a cutoff point of 0.368, sensitivity value of 0.920, and specificity value of 0.809 (Figure 3(c), Supplementary Table 6). The model consisting of four variables (BMI, FBG, testosterone, and DIAPH1) predicted PCOS with a sensitivity of 92% and a specificity of 80.9% (Figure 3(c), Supplementary Table 6).

BMI, FBG, testosterone, and DIAPH1 were all significantly associated with PCOS in univariate analyses (Table 3). Multivariate analysis using these variables showed that DIAPH1 was independently associated with PCOS (OR: 0.15, 95% CI: 0.05–0.39; *P* < 0.001; Table 3). Therefore, the plasma DIAPH1 level could also be an independent risk factor for PCOS. A nomogram for predicting PCOS risk was constructed using the variables in model C (Figure 3(e)). For example, for evaluating the risk of PCOS, a woman with BMI 26 kg/m^2^, plasma DIAPH1 level 2.5 mg/mL, FBG 7 mmol/L, and testosterone 1.5 nmol/L was predicted to have a probability of PCOS of approximately 88%.

## 7. Discussion

In the current study, we investigated the plasma DIAPH1 levels in women with PCOS compared to healthy control women and in BMI-divided NW or OW subgroups. The correlation of DIAPH1 with glucose and insulin metabolic indicators and lipid and inflammatory markers was analyzed. The dominant findings of this study were that the plasma DIAPH1 level was associated with FBG, HOMA-*β*, TG, and LH/FSH levels. DIAPH1 combined with testosterone, BMI, and FBG was the preferable predictive model of PCOS.

The role of DIAPH1 in systemic metabolism was recognized recently, but few investigations refer to the functions of DIAPH in PCOS and circulating proteomics. Several studies have emphasized the role of DIAPH1 in RAGE signal transduction, which is related to diabetes and obesity. For example, the RAGE-DIAPH1 axis contributes to inflammatory signaling in diabetes-associated nephropathy [17]. AGEs are also involved in islet *β*-cell damage in diabetes partially via RAGEs [25]. The gene expressions of the AGE/RAGE/DIAPH1 axis is positively associated with inflammatory and adipogenic markers in subcutaneous adipose tissue [26]. DIAPH1 is also a key regulator of steroid hormones and adrenal androgen production because it mediates ACTH-stimulated cortisol biosynthesis by coordinating dynamic mitochondrial trafficking [18]. DIAPH1 interacts with tubulin, actin, and vimentin in human adrenocortical cells, which may facilitate the efficient transport of substrates during steroid hormone production [19]. However, whether DIAPH1 contributes to the development of metabolic disturbance in PCOS patients is not known. Because DIAPH1-mediated RAGE signal transduction has multiple functions in glucose metabolism, inflammation and IR in PCOS and DIAPH1 also regulates hormone metabolism, we hypothesized that DIAPH1 was involved in these pathological processes in PCOS patients.

Our clinical observations showed that women with PCOS manifested classical symptoms, such as hyperandrogenism, and increased LH/FSH and IR, and 74.67% of women with PCOS were overweight or obese, without filtration during enrollment, which is consistent with the increased prevalence of metabolic syndrome in women with PCOS. We found that PCOS patients showed inordinate glucose and insulin indicators and higher UA, which were reported previously [27, 28]. Lipid metabolic disorders and inflammation were aggravated in OW-PCOS patients, who had dyslipidemia, abnormal liver function, and higher WBCs than their BMI-matched control NW-PCOS patients did not exhibit these changes. DIAPH1 closely participates in RAGE signal transduction, which is responsible for glucose and lipid metabolites. Therefore, the elevated plasma DIAPH1 levels in OW women suggest a threat of hyperglycemia and obesity. Notably, the DIAPH1 levels decreased in OW participants when they had PCOS. This relationship might suggest a protective role of reduced DIAPH1 levels in PCOS patients, which also displays the complexity of PCOS, a syndrome of metabolic disorders and hormone dysregulation. For example, the complexity reflected that DIAPH1 levels positively correlated with glucose and lipid metabolism but negatively correlated with sex hormone indicators, as shown in Table 2. Thus, DIAPH1 change was a consequence of multiple pathological conditions in OW-PCOS patients. Compared to NW-PCOS patients, the OW-PCOS patients exhibited robustly increased OGTT 60 min and 120 min insulin, AUC levels of insulin, decreased SHBG, and increased FAI, which is consistent with previous clinical observations. These results suggest a delayed peak of insulin secretion, IR, suppressed binding of SHBG with testosterone, and elevated free androgen. Notably, the correlation between DIAPH1, glucose metabolism, and IR in PCOS was certain because DIAPH1 levels always significantly correlated with FBG and HOMA-*β* levels in all participants, including overweight/obese and PCOS women.

Glucose intolerance and hyperinsulinemia are important metabolic features of PCOS pathogenesis [1, 3, 19]. Therefore, we analyzed the differences in DIAPH1 levels in different FBG or HOMA-*β* subgroups of PCOS patients. We found that an increased circulating DIAPH1 level was more common in the higher FBG groups and lower HOMA-*β* groups. This discordance suggests that glucose intolerance and insulin secretion disorder play an integrated role in DIAPH1 levels. Notably, TC levels differently changed in the DIAPH1 quantile groups of PCOS women and positively correlated with DIAPH1 in the total and OW participants. Although differences in sex hormones and inflammatory indicators were not readily apparent in the DIAPH1 quantile groups of PCOS women, the levels of testosterone, estrogen, progesterone, and LH/FSH were significantly associated with DIAPH1 in OW women. TNF-*α* was associated with DIAPH1 in PCOS women. Chronic inflammation due to the abnormal production of cytokines and activation of inflammatory signaling pathways is closely associated with metabolic disorders, such as obesity, insulin resistance, T2DM, and PCOS [29]. Subclinical chronic inflammation status existed in PCOS patients. TNF-*α* is an important inflammatory mediator released by adipocytes and inflammatory cells that reflect the insulin resistance state [30]. Other studies showed that gene expression of the AGE/RAGE/DIAPH1 axis was strongly and positively associated with inflammatory and adipogenic markers and HOMA-IR in subcutaneous adipose tissue [24]. DIAPH1 also participates in the steroid hormone biosynthetic pathway and adrenal androgen secretion [17]. Our findings suggested that plasma DIAPH1 was related to glucose metabolism and synthetically influenced by body fat content and sex hormones, which echoed the previously demonstrated connection of DIAPH1 with inflammatory and adipogenic markers and hormones [19, 26]. Several inflammatory molecules, such as TNF-*α*, IL-6, NF-*κ*B, and miR-223-3p, were investigated as pivotal regulators of inflammation and insulin pathways in PCOS [31, 32]. Anti-inflammatory supplements, such as nanocurcumin, ameliorated the inflammatory state of PCOS [31, 32]. Therefore, further research is required about the possible mechanism and therapeutic use of DIAPH1 against PCOS pathologies.

Univariate analysis and the clinical importance supported the final diagnostic model that included BMI, FBG, testosterone, and DIAPH1. The present study showed that decreased concentrations of plasma DIAPH1 were independent predictors of PCOS risk in a model in combination with BMI, FBG, and testosterone (92.0% sensitivity and 80.9% specificity). The sensitivity is useful in this case. Therefore, these findings provide references to evaluate the risk of having PCOS in women of child-bearing age.

Unfortunately, our investigation is limited by the observational design. However, this study first demonstrated the potential correlations between plasma DIAPH1 and metabolic or sexual parameters in a limited number of participants. Validation of the specificity of our results is needed, likely via the evaluation of DIAPH1 changes in a series of female reproductive disorders and diabetes or obesity patients. To investigate the precise mechanisms, more detailed *in vivo* and *in vitro* experiments are needed in future studies.

## 8. Conclusions

The present study analyzed the potential roles of plasma DIAPH1 in women with PCOS and control participants and demonstrated that plasma DIAPH1 levels decreased in women with PCOS and were associated with glucose metabolism, IR, body fat, inflammation, and sex hormone metabolism. DIAPH1 is an independent risk factor, and the model containing DIAPH, BMI, FBG, and testosterone may be a predictor for PCOS occurrence. Further studies are needed to elucidate the mechanism of DIAPH1 in the pathogenesis and development of PCOS.

## Figures and Tables

**Table 1:** Clinical parameters of the study participants.

	Normal range	Control			PCOS			*P* value
		Total (*n* = 77)	Con-NW (*n* = 47)	Con-OW (*n* = 30)	Total (*n* = 75)	PCOS-NW (*n* = 19)	PCOS-OW (*n* = 56)	(Total PCOS vs. total con)
Age (y)	—	27 (24, 30)	27 (23.5, 31.5)	27 (24.25, 30)	26 (21, 28.5)	25 (20.5, 29.5)	26 (21, 28)	0.032
BMI (kg/m^2^)	18.5-24	23.05 (21.26, 24.56)	21.5 (20.3, 22.69)	25.46 (24.44, 26.02)^a^	25.65 (23.83, 28.85)	22.86 (19.74, 23.33)	27.58 (25.3, 30.73)^c,d^	<0.001
Waist (cm)	<88	78 (70.6, 81)	74 (68.8, 77.8)	83.95 (80.5, 88.5)^a^	89 (82.5, 99.5)	80 (76, 85) b	95.25 (87.75, 102)^c,d^	<0.001
WHR	<0.86	0.87 (0.83, 0.89)	0.86 (0.8, 0.88)	0.89 (0.87, 0.91)^a^	0.89 (0.87, 0.94)	0.86 (0.83, 0.89)	0.9 (0.87, 0.95)^c,d^	<0.001
SBP (mmHg)	90-140	112 (106, 121)	113 (107, 120.5)	111 (106, 120.75)	117 (108.5, 128.5)	112 (106.5, 117.5)	120.5 (109, 132)^c^	0.043
DBP (mmHg)	60-90	69.97 ± 8.4	70.91 ± 7.13	68.5 ± 10.02	76.15 ± 8.65	71.58 ± 7.83	77.7 ± 8.42^c,d^	<0.001
FBG (mmol/L)	3.9-6.1	4.9 (4.6, 5.3)	4.82 (4.39, 5.02)	5.2 (4.78, 5.43)^a^	4.89 (4.57, 5.38)	4.65 (4.48, 4.79)	4.97 (4.74, 5.54)^c^	0.475
FINS (mU/mL)	1.5-15	7.6 (5.9, 10.2)	6.9 (5.35, 8.3)	10.4 (8, 11.93)^a^	14.8 (11.05, 18.35)	9.3 (4.25, 11.7) b	16.65 (13.15, 19.45)^c,d^	<0.001
HbA1c (%)	4-6%	5.1 (5, 5.45)	5.1 (5, 5.7)	5 (5, 5.1)^a^	5.5 (5.2, 5.8)	5.3 (5.05, 5.4) b	5.6 (5.3, 6.03)^c,d^	<0.001
HOMA-IR	≤1.6	1.65 (1.33, 2.35)	1.44 (1.12, 1.72)	2.46 (1.83, 2.75)^a^	3.31 (2.4, 4.27)	1.85 (0.88, 2.61) b	3.9 (2.97, 4.74)^c,d^	<0.001
HOMA-*β*	100%	112.71 (82.61, 162.5)	110.34 (81.49, 159.52)	127.34 (87.98, 166.43)	186.32 (124.62, 262.79)	124.59 (67.99, 191.84)	214.9 (144.97, 272.29)^c,d^	<0.001
TG (mmol/L)	0-1.7	0.94 (0.78, 1.27)	0.92 (0.76, 1.17)	1.04 (0.81, 1.38)	1.48 (1.1, 2.12)	0.89 (0.7, 1.5)	1.67 (1.23, 2.23)^c,d^	<0.001
TC (mmol/L)	0-5.2	4.3 (3.86, 4.88)	4.11 (3.78, 4.81)	4.57 (3.94, 5.15)	4.45 (4.02, 5.03)	4.39 (3.85, 4.84)	4.53 (4.04, 5.15)	0.345
HDL-C (mmol/L)	0.9-1.68	1.59 (1.31, 2)	1.72 (1.27, 2)	1.56 (1.45, 1.77)	1.15 (0.96, 1.32)	1.29 (1.08, 1.79)	1.08 (0.93, 1.25)^d^	<0.001
LDL-C (mmol/L)	2.07-3.1	2.34 ± 0.75	2.32 ± 0.77	2.37 ± 0.73	2.69 ± 0.73	2.54 ± 0.63	2.74 ± 0.76	0.004
ALT (IU/L)	7-40	15 (11, 21.6)	13.9 (10.85, 19.05)	15.6 (12.62, 29.75)	24.6 (14, 48)	13 (10.45, 17.2)	37.15 (17.45, 62.92)^c,d^	<0.001
AST (IU/L)	13-35	18.3 (15, 22)	19 (15.45, 22)	17.25 (15.03, 21.75)	21 (16.15, 28.3)	17.4 (14.1, 19.3)	22.1 (17.05, 37.73)^c,d^	0.042
*γ*-GGT (IU/L)	7-45	15.9 (12.9, 19)	14 (11.95, 18.2)	17.5 (15.22, 19.75)^a^	30.2 (16.75, 55.05)	17 (11.8, 29.9)	39.25 (22.82, 57.05)^c,d^	<0.001
UA (*μ*mol/L)	89~ 357	265 (237.2, 300)	245.8 (227.65, 275.3)	298.95 (277.2, 342.75)^a^	401.3 (302.7, 455.85)	313.7 (280.05, 353.8) b	409.65 (355.05, 469.15)^c,d^	<0.001
Creatinine (*μ*mol/L)	45-105	54.42 ± 7.24	54.25 ± 6.8	54.68 ± 7.99	58.96 ± 8.46	58.44 ± 4.99	59.13 ± 9.38	<0.001
BUN (mmol/L)	2.9-8.2	4.3 (3.6, 5.04)	4.4 (3.9, 5.06)	4.23 (3.52, 4.86)	4.35 (3.63, 5.2)	4.39 (3.78, 5.22)	4.34 (3.58, 5.19)	0.874
Testosterone (nmol/L)	0.38-1.97	0.9 (0.58, 1.29)	0.64 (0.5, 0.85)	1.25 (1.08, 1.62)^a^	2.11 (1.71, 2.68)	1.99 (1.69, 2.45)^b^	2.14 (1.75, 2.71)^d^	<0.001
LH (mIU/L)	Follicular phase: 2.39-6.6	4.7 (3.31, 6.99)	5.36 (4.46, 6.77)	3.87 (2.17, 7.18)	8.5 (5.18, 10.62)	8.12 (5.08, 10.3)^b^	8.59 (5.36, 10.61)^d^	<0.001
FSH (mIU/L)	Follicular phase: 3.03-8.08	4.41 (2.97, 6.04)	4.39 (3.04, 6.35)	4.78 (3.11, 5.79)	4.06 (3.44, 4.82)	4.31 (3.1, 4.88)	4.01 (3.47, 4.82)	0.123
LH/FSH	<2	1.06 (0.71, 1.61)	1.11 (0.86, 1.68)	0.83 (0.59, 1.35)^a^	2.02 (1.45, 2.55)	1.76 (1.42, 2.41)^b^	2.03 (1.56, 2.6)^d^	<0.001
Estradiol (pg/mL)	Follicular phase: 21-251	78.46 (54.75, 151.32)	94.69 (63.47, 161.56)	71.5 (37.25, 123)	51 (34, 70.5)	46 (21.5, 58)^b^	52.5 (38.25, 73.75)	<0.001
Progesterone (ng/mL)	Follicular phase: <0.1-0.3	1.33 (0.5, 9.35)	1.59 (0.88, 11.59)	0.55 (0.4, 7.72)^a^	0.4 (0.2, 0.63)	0.4 (0.3, 0.63)^b^	0.4 (0.2, 0.62)^c,d^	<0.001
PRL (ng/mL)	5.18-26.53	16.3 (10.13, 26.48)	15.45 (10.27, 23.85)	17.64 (10.19, 28.71)	16.62 (9.72, 22.9)	13.35 (9.35, 19.67)	17.12 (9.74, 23.62)	0.324
WBC (∗10^9/L)	3.5-9.5	5.73 (4.9, 6.42)	5.67 (4.86, 6.34)	5.8 (5.14, 6.46)	6.55 (5.72, 7.92)	5.7 (4.94, 6.43)	6.97 (6.11, 8.19)^c,d^	<0.001
DIAPH1 (ng/mL)	—	3.05 (2.74, 3.55)	2.81 (2.64, 3.07)	3.79 (3.15, 3.97)^a^	2.78 (2.46, 3.28)	3.14 (2.61, 3.51)	2.69 (2.43, 3.18)^d^	0.01

Data are shown as the mean ± SD for variables with a normal distribution and median with the interquartile range (25–75%) for nonnormally distributed continuous variables. For variables with a normal distribution, an independent samples *t*-test was performed to compare variables between two groups; a one-way ANOVA followed by Tukey multiple comparison test was performed among the four subgroups (Con-NW, Con-OW, PCOS-NW, and PCOS-OW). For nonnormally distributed continuous variables, the Mann–Whitney *U* test was performed to compare variables between the two groups; the Kruskal-Wallis test followed by pairwise comparisons using BWS all-pairs test was performed for the four subgroups. Con-NW: control normal-weight participants; Con-OW: control overweight/obese participants; PCOS-NW: PCOS normal-weight participants; PCOS-OW: PCOS overweight/obese participants; BMI: body mass index; WHR: waist-to-hip ratio; SBP: systolic blood pressure; DBP: diastolic blood pressure; FBG: fasting blood glucose; FINS: fasting insulin; HbA1c: hemoglobin A1c; HOMA-IR: homeostatic model assessment for insulin resistance; HOMA-*β*: homoeostasis model assessment of insulin resistance and insulin secretion; TC: total cholesterol; TG: triglycerides; LDL-C: low-density lipoprotein cholesterol; HDL-C: high-density lipoprotein cholesterol; AST: aspartate transaminase; ALT: alanine transaminase; *γ*-GGT: gamma-glutamyl transpeptidase; UA: uric acid; BUN: blood urea nitrogen; PCOS: polycystic ovary syndrome; FSH: follicle-stimulating hormone; LH: luteinizing hormone; PRL: prolactin; WBC: white blood cell count. a *P* < 0.05 for NW-Con vs. OW-Con; b *P* < 0.05 for NW-Con vs NW-PCOS; c *P* < 0.05 for NW-PCOS vs. OW-PCOS; d *P* < 0.05 for OW-Con vs. OW-PCOS.

**Table 2:** The correlations between clinical indicators and DIAPH1.

	Total		OW		PCOS	
	*r*	*P* value	*r*	*P* value	*r*	*P* value
Age (y)	0.021	0.798	0.206	0.057	0.119	0.308
BMI (kg/m^2^)	0.104	0.202	-0.077	0.481	-0.083	0.479
WHR	0.162	0.047	0.119	0.275	0.075	0.524
FBG (mmol/L)	0.351	<0.0001	0.379	< 0.001	0.464	<0.0001
FINS (mU/mL)	-0.021	0.799	-0.207	0.056	-0.112	0.337
HbA1c (%)	0.034	0.679	0.023	0.834	0.281	0.015
HOMAIR	0.067	0.412	-0.090	0.412	0.040	0.733
HOMA-*β*	-0.211	0.009	-0.371	< 0.001	-0.327	0.004
TG (mmol/L)	0.101	0.217	0.017	0.874	0.155	0.185
TC (mmol/L)	0.178	0.029	0.228	0.035	0.184	0.115
HDL-C (mmol/L)	0.034	0.680	0.123	0.258	-0.036	0.761
LDL-C (mmol/L)	0.033	0.685	0.030	0.787	0.109	0.352
ALT (IU/L)	0.071	0.386	-0.048	0.664	0.082	0.484
AST (IU/L)	0.070	0.389	0.027	0.805	0.080	0.494
*γ*-GGT (IU/L)	0.124	0.128	-0.007	0.948	0.312	0.006
UA (*μ*mol/L)	-0.033	0.687	-0.277	0.010	-0.038	0.743
Creatinine (*μ*mol/L)	-0.099	0.224	-0.152	0.162	0.033	0.779
BUN (mmol/L)	-0.095	0.245	0.029	0.788	-0.026	0.823
Testosterone (nmol/L)	-0.053	0.526	-0.234	0.030	-0.054	0.643
LH (mIU/L)	-0.106	0.210	-0.109	0.318	-0.066	0.572
FSH (mIU/L)	0.132	0.117	0.145	0.182	0.155	0.185
LH/FSH	-0.172	0.040	-0.243	0.024	-0.082	0.483
Estradiol (pg/mL)	0.117	0.163	0.345	0.001	0.033	0.781
Progesterone (ng/mL)	0.125	0.137	0.268	0.013	0.022	0.850
PRL (ng/mL)	-0.013	0.873	-0.028	0.799	-0.020	0.867
WBC (∗10^9/L)	-0.048	0.557	-0.129	0.239	0.038	0.746

The correlations were determined by the Spearman analysis. Total: all participants; OW: overweight participants; PCOS: participants with polycystic ovary syndrome.

**Table 3:** Univariate and multivariable logistic regression analyses of associations between clinical and biochemical variables and PCOS.

Variable	Univariate analysis		Multivariate analysis		
	OR (95% CI)	*P* value	*β*	OR (95% CI)	*P* value
BMI	1.28 [1.16, 1.44]	<0.001	0.16	1.17 [1.03, 1.36]	0.026
FBG	1.63 [1.05, 2.69]	0.041	0.51	1.67 [0.80, 3.72]	0.182
Testosterone	9.19 [4.78, 19.92]	<0.001	2.26	9.58 [4.63, 23.02]	<0.001
DIAPH1	0.55 [0.31, 0.93]	0.029	-1.56	0.21 [0.08, 0.49]	0.001

OR: odd ratio; CI: confidence interval; *β* is the regression coefficient.

## Data Availability

The statistical data used to support the findings of this study are available from the corresponding author upon request.

## Supplementary Materials

Supplementary Materials Supplementary Table 1: supplementary characteristics of OGTT insulin, sex-related hormones, and inflammatory indicators in NW and OW PCOS patients. Supplementary Figure 1: the correlation of plasma DIAPH1 levels with levels of FBG, HbA1c, HOMA-*β*, LH/FSH, *γ*-GGT, and TNF-*α* in PCOS patients. Supplementary Table 2: correlation of DIAPH1 with OGTT insulin, sex-related hormones, and inflammatory indicators in PCOS patients. Supplementary Table 3: clinical characteristics and biochemical variables of different FBG in PCOS patients. Supplementary Table 4: clinical characteristics and biochemical variables of different HOMA-*β* in PCOS patients. Supplementary Table 5: clinical characteristics and biochemical variables in PCOS patients of different DIAPH1 quartiles. Supplementary Table 6: construction of regression models containing vital clinical indicators and DIAPH1. Supplementary Table 7: the test method and equipment of clinical indicators.
