# Supplementary material for: Plasma Diaphanous Related Formin 1 Levels Are Associated with Altered Glucose Metabolism and Insulin Resistance in Patients with Polycystic Ovary Syndrome: A Case Control Study
Source: Mediators Inflamm. 2022 Feb 11;2022:9620423. doi: 10.1155/2022/9620423 (PMC8856793; doi:10.1155/2022/9620423)
Supplement: Supplementary Materials — Supplementary Table 1: supplementary characteristics of OGTT insulin, sex-related hormones, and inflammatory indicators in NW and OW PCOS patients. Supplementary Figure 1: the correlation of plasma DIAPH1 levels with levels of FBG, HbA1c, HOMA-β, LH/FSH, γ-GGT, and TNF-α in PCOS patients. Supplementary Table 2: correlation of DIAPH1 with OGTT insulin, sex-related hormones, and inflammatory indicators in PCOS patients. Supplementary Table 3: clinical characteristics and biochemical variables of different FBG in PCOS patients. Supplementary Table 4: clinical characteristics and biochemical variables of different HOMA-β in PCOS patients. Supplementary Table 5: clinical characteristics and biochemical variables in PCOS patients of different DIAPH1 quartiles. Supplementary Table 6: construction of regression models containing vital clinical indicators and DIAPH1. Supplementary Table 7: the test method and equipment of clinical indicators. [file 9620423.f1.docx]

Supplementary Table 1. Supplementary characteristics of OGTT insulin, sex-related hormones and inflammatory indicators in NW and OW PCOS patients.

|  | Normal range | PCOS-NW  (n = 19) | PCOS-OW  (n = 56) | *P* value |
| --- | --- | --- | --- | --- |
| OGTT 30min insulin (mU/mL) | 25-85uU/mL | 78.95 (53.25, 89.88) | 89 (62.4, 122.6) | 0.145 |
| OGTT 60min insulin (mU/mL) | 15-50uU/mL | 58.5 (45.4, 72.32) | 97.25 (64.03, 148.75)^a^ | 0.001 |
| OGTT 120min insulin (mU/mL) | 15-40uU/mL | 45.75 (24.18, 78.38) | 92.1 (63.95, 137.88)^a^ | 0.001 |
| OGTT 180min insulin (mU/mL) | 5-17uU/mL | 33.7 (12.32, 69.15) | 45.8 (27.92, 83.35) | 0.116 |
| AUC-Insulin | - | 207.57 (167.66, 274.65) | 353.62 (231.78, 454.36)^a^ | < 0.001 |
| SHBG (nmol/L) | 34.3-147.7nmol/L | 68.1 (55.55, 133) | 24.5 (14.55, 38.83)^a^ | < 0.001 |
| DHEA-S (μg/dl) | 238.4-539.3μg/dL | 310.2 (227.6, 324.6) | 376.75 (264.7, 526.38) | 0.05 |
| FAI | 7-10 | 2.27 (1.52, 4.14) | 9.08 (5.18, 14.52)^a^ | < 0.001 |
| CRP (mg/L) | 0-8mg/L | 5 (2.4, 6.35) | 4.6 (2.73, 5.59) | 0.909 |
| IL-6 (pg/mL) | 0-3.4pg/mL | 2.8 (2, 4.4) | 4.4 (2.4, 8.1) | 0.102 |
| TNF-α (pg/mL) | 0-8.1pg/mL | 6.45 (5.43, 8.5) | 8 (6.9, 10.4) | 0.132 |
| IL-8 (pg/mL) | ＜62pg/mL | 8.92 (6.84, 12.6) | 8.75 (5.23, 18.9) | 0.833 |

Data are presented as interquartile range (25–75%). Mann-Whitney U test was performed to compare variables between two groups. a *P* < 0.05. OGTT: oral glucose tolerance test; AUC-Insulin, area under the curve for insulin; SHBG, sex hormone-binding globulin; DHEA-S: dehydroepiandrosterone sulfate; FAI: free androgen index; CRP: C-reactive protein; IL-6: Interleukin-6; TNF-α: tumor necrosis factor alpha.


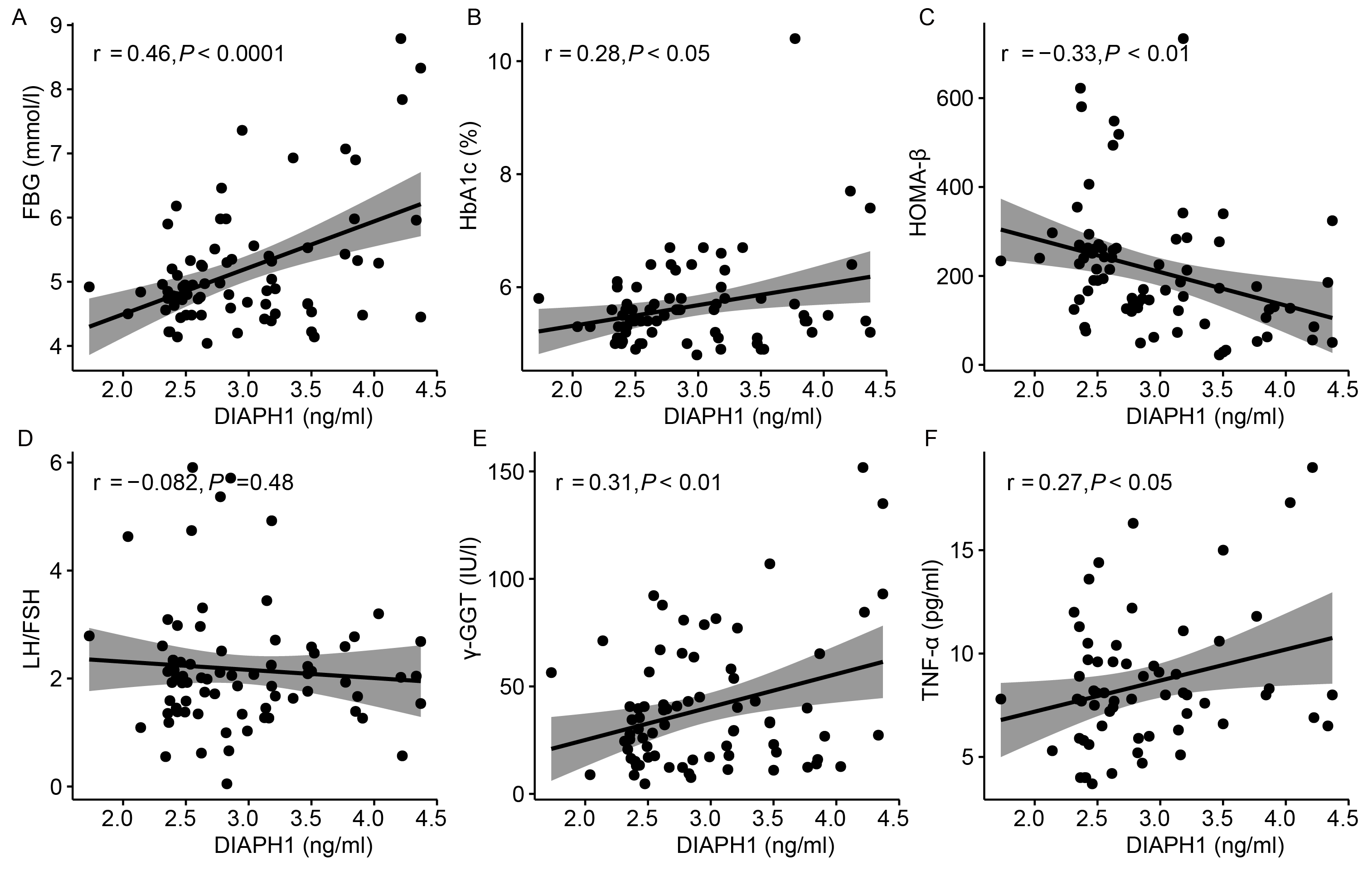


Supplementary Figure 1. The correlation of plasma DIAPH1 levels with levels of FBG, HbA1c, HOMA-β, LH/FSH, γ-GGT and TNF-α in PCOS patients. The correlations were determined by the Spearman correlation analysis. FBG: fasting blood glucose; HbA1c: hemoglobin A1c; HOMA-β, homoeostasis model assessment of insulin secretion; γ-GGT: gamma-glutamyl transpeptidase; FSH: follicle- stimulating hormone; LH: luteinizing hormone; TNF-α: tumor necrosis factor alpha.

Supplementary Table 2. Correlation of DIAPH1 with OGTT insulin, sex-related hormones and inflammatory indicators in PCOS patients.

|  | PCOS | |
| --- | --- | --- |
|  | *r* | *P* value |
| OGTT 30min insulin (mU/mL) | -0.165 | 0.180 |
| OGTT 60min insulin (mU/mL) | -0.101 | 0.407 |
| OGTT 120min insulin (mU/mL) | -0.075 | 0.541 |
| OGTT 180min insulin (mU/mL) | 0.163 | 0.180 |
| AUC-Insulin | -0.099 | 0.417 |
| SHBG (nmol/L) | 0.188 | 0.134 |
| DHEA-S (μg/dl) | -0.057 | 0.653 |
| FAI | -0.021 | 0.868 |
| CRP (mg/L) | 0.179 | 0.154 |
| IL-6 (pg/mL) | -0.056 | 0.665 |
| TNF-α (pg/mL) | **0.272** | **0.034** |
| IL-8 (pg/mL) | -0.078 | 0.549 |

The correlations were determined by the Spearman analysis.

Supplementary table 3. Clinical characteristics and biochemical variables of different FBG in PCOS patients.

|  | PCOS | |  |
| --- | --- | --- | --- |
|  | Relative-low FBG  (n = 45) | Relative-high FBG  (n = 30) | *P* value |
| Age (y) | 26 (21, 28) | 25.5 (21, 30.75) | 0.543 |
| BMI (kg/m2) | 24.68 (23.23, 27.78) | 27.61 (25.29, 30.49) | 0.015 |
| Waist (cm) | 87.89 ± 10.58 | 94.85 ± 11.83 | 0.012 |
| WHR | 0.89 ± 0.06 | 0.91 ± 0.08 | 0.163 |
| SBP (mmHg) | 116.11 ± 13.97 | 121.8 ± 14.63 | 0.098 |
| DBP (mmHg) | 74.18 ± 7.62 | 79.1 ± 9.38 | 0.02 |
| FBG (mmol/L) | 4.65 (4.48, 4.8) | 5.54 (5.32, 6.39) | < 0.001 |
| FINS (mU/mL) | 13.2 (8.3, 16.7) | 17.15 (12.82, 21.98) | 0.001 |
| HbA1c (%) | 5.4 (5.1, 5.6) | 5.65 (5.4, 6.4) | 0.004 |
| HOMAIR | 2.55 (1.79, 3.54) | 4.57 (3.56, 5.76) | < 0.001 |
| HOMA-β | 227.42 (130.61, 282.61) | 148.33 (110.99, 186.08) | 0.032 |
| OGTT 30min insulin (mU/mL) | 85.65 (66.92, 112.25) | 82.1 (46.5, 122.6) | 0.488 |
| OGTT 60min insulin (mU/mL) | 75.4 (54, 105) | 97.1 (67, 146.8) | 0.117 |
| OGTT 120min insulin (mU/mL) | 74 (45.5, 107.2) | 95.9 (60.9, 174.1) | 0.041 |
| OGTT 180min insulin (mU/mL) | 37.5 (18.5, 67.3) | 51.8 (37.2, 92.7) | 0.025 |
| AUCI | 269.3 (205.35, 377.6) | 356.35 (232, 431.4) | 0.11 |
| TG (mmol/L) | 1.38 (0.89, 1.75) | 1.75 (1.21, 2.36) | 0.054 |
| TC (mmol/L) | 4.46 ± 0.71 | 4.7 ± 0.96 | 0.263 |
| HDL-C (mmol/L) | 1.18 (0.99, 1.41) | 1.1 (0.94, 1.2) | 0.2 |
| LDL-C (mmol/L) | 2.58 ± 0.64 | 2.84 ± 0.84 | 0.158 |
| ALT (IU/L) | 22.2 (13, 40) | 41.2 (15.93, 58.2) | 0.067 |
| AST (IU/L) | 19.1 (15.3, 23.3) | 21.9 (17.12, 38.8) | 0.137 |
| γ-GGT (IU/L) | 26 (17, 40.6) | 40.35 (18.82, 64.8) | 0.127 |
| UA (μmol/L) | 373.16 ± 91.07 | 413.25 ± 112.81 | 0.11 |
| Creatinine (μmol/L) | 59.08 ± 7.4 | 58.78 ± 9.97 | 0.887 |
| BUN (mmol/L) | 4.52 (3.62, 5.18) | 4.34 (3.7, 5.06) | 0.713 |
| Testosterone (nmol/L) | 2.06 (1.73, 2.67) | 2.26 (1.65, 2.7) | 0.513 |
| FSH (mIU/L) | 4.26 (3.41, 4.82) | 4 (3.47, 4.76) | 0.867 |
| LH (mIU/L) | 8.93 (5.11, 10.91) | 8.26 (6.72, 9.91) | 0.944 |
| Estradiol (pg/mL) | 51 (31, 64) | 51 (35.25, 78.25) | 0.452 |
| Progesterone (ng/mL) | 0.4 (0.2, 0.7) | 0.4 (0.2, 0.58) | 0.718 |
| PRL (ng/mL) | 19 (12.06, 24.34) | 10.63 (8.44, 17.86) | 0.007 |
| LH/FSH | 1.93 (1.38, 2.47) | 2.03 (1.68, 2.57) | 0.607 |
| SHBG (nmol/L) | 35 (23.8, 63.1) | 22.5 (11.65, 37.47) | 0.01 |
| DHEA-S (μg/dl) | 372.19 ± 182.96 | 391.91 ± 197.33 | 0.682 |
| FAI | 5.98 (2.27, 11.16) | 9.28 (5.17, 17.98) | 0.03 |
| CRP (mg/L) | 4.8 (2.92, 5.47) | 4.6 (2.3, 7.4) | 0.973 |
| IL-6 (pg/mL) | 4.65 (2.17, 8.65) | 4.05 (2.3, 6.88) | 0.498 |
| TNF-α (pg/mL) | 7.8 (6, 9.1) | 8.05 (6.8, 10.72) | 0.216 |
| IL-8 (pg/mL) | 7.88 (5.16, 12.57) | 8.83 (5.6, 22.67) | 0.482 |
| WBC (*10^9/L) | 6.29 ± 1.33 | 7.44 ± 1.89 | 0.006 |
| DIAPH1 (ng/mL) | 2.6 (2.41, 3.14) | 3.1 (2.74, 3.82) | 0.002 |

Data are shown as mean ± SD for variables of normal distribution, and median with the interquartile range (25-75%) for skewed variables. For variables of normal distribution, independent sample t-test was performed to compare variables between two groups. For variables of skewed distribution, Mann-Whitney U test was performed to compare variables between two groups. Relative-low FBG means FBS < 5 mmol/L; relative-high FBG means FBS ≥ 5 mmol/L.

Supplementary table 4. Clinical characteristics and biochemical variables of different HOMA-β in PCOS patients.

|  | PCOS | |  |
| --- | --- | --- | --- |
|  | Relative-low HOMA-β  (n = 41) | Relative-high HOMA-β  (n = 34) | *P* value |
| Age (y) | 26 (24, 30) | 25.5 (19.25, 28) | 0.106 |
| BMI (kg/m2) | 25.28 (23.15, 27.74) | 27.11 (24.79, 31.82) | 0.016 |
| Wasist (cm) | 88.88 ± 11.58 | 92.84 ± 11.28 | 0.139 |
| WHR | 0.9 ± 0.08 | 0.9 ± 0.06 | 0.778 |
| SBP (mmHg) | 117.51 ± 14.54 | 119.44 ± 14.4 | 0.567 |
| DBP (mmHg) | 77.46 ± 9.35 | 74.56 ± 7.56 | 0.141 |
| FBG (mmol/L) | 5.32 (4.66, 5.98) | 4.76 (4.48, 4.95) | < 0.001 |
| FINS (mU/mL) | 11.4 (8.1, 15.8) | 17.55 (14.43, 20.77) | < 0.001 |
| HbA1c (%) | 5.5 (5.2, 6.3) | 5.4 (5.12, 5.7) | 0.144 |
| HOMAIR | 2.95 (1.65, 4.25) | 3.69 (2.97, 4.28) | 0.021 |
| HOMA-β | 126.37 (76.11, 153.85) | 270.2 (241.64, 341.13) | < 0.001 |
| OGTT 30min insulin (mU/mL) | 70 (46.5, 87.9) | 107.2 (76.85, 127.23) | 0.001 |
| OGTT 60min insulin (mU/mL) | 67.5 (52.4, 97.1) | 102 (68.8, 160.5) | 0.014 |
| OGTT 120min insulin (mU/mL) | 65.1 (41.7, 110.2) | 98.6 (69.6, 135) | 0.045 |
| OGTT 180min insulin (mU/mL) | 38.6 (21.3, 59.7) | 53.3 (34.3, 83.2) | 0.167 |
| AUCI | 215.15 (173.6, 359.4) | 356.35 (266.25, 464.55) | 0.002 |
| TG (mmol/L) | 1.42 (1.08, 2.12) | 1.56 (1.21, 2.13) | 0.36 |
| TC (mmol/L) | 4.81 ± 0.89 | 4.25 ± 0.61 | 0.002 |
| HDL-C (mmol/L) | 1.18 (1.03, 1.41) | 1.08 (0.93, 1.22) | 0.271 |
| LDL-C (mmol/L) | 2.94 ± 0.72 | 2.38 ± 0.64 | < 0.001 |
| ALT (IU/L) | 19.6 (12.3, 48.2) | 29.7 (16.75, 46.67) | 0.377 |
| AST (IU/L) | 19.5 (16.2, 31.2) | 21.05 (15.63, 24.88) | 0.721 |
| γ-GGT (IU/L) | 29.5 (15.1, 58.1) | 33.3 (21.02, 50.65) | 0.786 |
| UA (μmol/L) | 383.6 ± 113.41 | 395.94 ± 86.27 | 0.594 |
| Creatinine (μmol/L) | 58.72 ± 8.38 | 59.25 ± 8.67 | 0.789 |
| BUN (mmol/L) | 4.39 (3.68, 5.67) | 4.16 (3.57, 4.75) | 0.338 |
| Testosterone (nmol/L) | 2.04 (1.68, 2.62) | 2.19 (1.84, 2.69) | 0.466 |
| FSH (mIU/L) | 4.26 (3.47, 5) | 3.92 (3.46, 4.61) | 0.25 |
| LH (mIU/L) | 8.71 (5.95, 11.01) | 8.35 (5.11, 10.36) | 0.397 |
| Estradiol (pg/mL) | 51 (35, 66) | 52.5 (33.5, 82.75) | 0.602 |
| Progesterone (ng/mL) | 0.4 (0.2, 0.6) | 0.4 (0.2, 0.65) | 0.944 |
| PRL (ng/mL) | 12.06 (8.71, 18.42) | 20.34 (13.43, 26.18) | 0.002 |
| LH/FSH | 2.04 (1.63, 2.51) | 1.96 (1.38, 2.61) | 0.659 |
| SHBG (nmol/L) | 33.9 (15.5, 62.43) | 24.9 (17.45, 38) | 0.365 |
| DHEA-S (μg/dl) | 365.44 ± 198.88 | 397.41 ± 177.08 | 0.495 |
| FAI | 5.6 (3.77, 11.33) | 9.59 (5.05, 13.23) | 0.193 |
| CRP (mg/L) | 4.6 (2.4, 5.6) | 4.75 (2.77, 5.45) | 0.963 |
| IL-6 (pg/mL) | 4.1 (2, 6.5) | 4.4 (3.2, 8.88) | 0.306 |
| TNF-α (pg/mL) | 8 (6.4, 10.4) | 7.8 (6.65, 9.07) | 0.507 |
| IL-8 (pg/mL) | 8.88 (6.69, 18.52) | 7.19 (5, 13.7) | 0.165 |
| WBC (*10^9/L) | 6.68 ± 1.87 | 6.84 ± 1.4 | 0.665 |
| DIAPH1 (ng/mL) | 3.04 (2.77, 3.77) | 2.54 (2.4, 2.91) | < 0.001 |

Data are shown as mean ± SD for variables of normal distribution, and median with the interquartile range (25-75%) for skewed variables. For variables of normal distribution, independent sample t-test was performed to compare variables between two groups. For variables of skewed distribution, Mann-Whitney U test was performed to compare variables between two groups. Relative-low HOMA-β (%) means HOMA-β (%) < 200, relative-high HOMA-β (%) means HOMA-β (%) ≥ 200.

Supplementary table 5. Clinical characteristics and biochemical variables in PCOS patients of different DIAPH1 quartiles.

|  | PCOS | | | |  |
| --- | --- | --- | --- | --- | --- |
| DIAPH1 quantile | Q1 (n = 18) | Q2 (n = 19) | Q3 (n = 19) | Q4 (n = 19) | *P* value |
| Age (y) | 24 (18.25, 28.75) | 26 (21.5, 28) | 27 (23.5, 29.5) | 25 (24, 29) | 0.445 |
| BMI (kg/m2) | 25.76 (24.24, 28.38) | 26.67 (24.94, 32.56) | 25.63 (23.79, 28.07) | 25.28 (22.9, 28.03) | 0.405 |
| Wasit (cm) | 89.31 ± 9.38 | 92.79 ± 10.07 | 90.63 ± 13.22 | 89.89 ± 13.46 | 0.813 |
| WHR | 0.88 ± 0.05 | 0.92 ± 0.08 | 0.88 ± 0.08 | 0.91 ± 0.07 | 0.17 |
| SBP (mmHg) | 119.83 ± 17.21 | 113.53 ± 10.68 | 121.11 ± 13.6 | 119.16 ± 15.49 | 0.387 |
| DBP (mmHg) | 75.06 ± 7.71 | 74.47 ± 6.2 | 77.63 ± 10.43 | 77.37 ± 9.82 | 0.595 |
| FBG (mmol/L) | 4.78 (4.58, 4.95) | 4.92 (4.6, 5.11) | 4.89 (4.62, 5.38) | 5.43 (4.59, 6.92) | 0.182 |
| FINS (mU/mL) | 17.15 (12.25, 20.27) | 15.6 (12.25, 18.95) | 14 (10.15, 15.8) | 12.2 (9.7, 16.4) | 0.16 |
| HbA1c (%) | 5.3 (5.12, 5.6) | 5.4 (5.4, 5.65) | 5.7 (5.4, 6.3) | 5.4 (5.15, 6.1) | 0.311 |
| HOMAIR | 3.72 (2.39, 4.26) | 3.43 (2.47, 4.54) | 2.97 (2.17, 4.08) | 3.28 (2.38, 4.69) | 0.848 |
| HOMA-β | 240 (151.6, 296.2) | 242.76 (192.02, 262.44) | 153.85 (134.11, 219.19) | 106.45 (54.31, 174.29)^b, d^ | 0.001 |
| OGTT 30min insulin (mU/mL) | 91 (82.1, 112.1) | 79.7 (66.48, 132.52) | 80.1 (63.45, 122.38) | 62.6 (44.33, 93.7) | 0.18 |
| OGTT 60min insulin (mU/mL) | 78.1 (66.8, 102) | 105 (71.12, 175.05) | 75.4 (57.75, 130.95) | 64.7 (52.77, 122.83) | 0.325 |
| OGTT 120min insulin (mU/mL) | 91.9 (65, 165.7) | 86.75 (68.45, 120.77) | 63.6 (37.6, 111.3) | 72.95 (57.48, 126.22) | 0.569 |
| OGTT 180min insulin (mU/mL) | 55.7 (17.1, 91.1) | 38.8 (26.42, 68.9) | 44 (24.25, 63.3) | 40.3 (30.52, 81.1) | 0.961 |
| AUC-Insulin | 297.9 (262.35, 429.5) | 365.7 (214.84, 461.34) | 280.35 (187.93, 383.75) | 247.63 (176.39, 360.03) | 0.421 |
| TG (mmol/L) | 1.35 (0.94, 1.67) | 1.51 (1.27, 2.08) | 1.61 (1.05, 2.12) | 1.51 (1.1, 2.5) | 0.534 |
| TC (mmol/L) | 4.23 ± 0.7 | 4.45 ± 0.7 | 4.96 ± 0.77^a^ | 4.57 ± 0.98 | 0.044 |
| HDL-C (mmol/L) | 1.14 (0.97, 1.38) | 1.2 (1, 1.31) | 1.18 (1.02, 1.66) | 1.06 (0.89, 1.15) | 0.358 |
| LDL-C (mmol/L) | 2.55 ± 0.74 | 2.64 ± 0.7 | 2.9 ± 0.79 | 2.65 ± 0.7 | 0.491 |
| ALT (IU/L) | 17.95 (14.3, 33.52) | 29.4 (16.05, 44.6) | 37.3 (15.3, 64.55) | 22.2 (13.35, 46.85) | 0.644 |
| AST (IU/L) | 18.5 (17.18, 22.35) | 19.5 (15.05, 23.45) | 23.2 (17.65, 39.5) | 21.2 (15.5, 28.25) | 0.448 |
| γ-GGT (IU/L) | 24.95 (15.1, 35.17) | 39 (19.85, 49.1) | 40.2 (17.5, 60.85) | 33 (17.7, 74.85) | 0.355 |
| UA (μmol/L) | 407.08 ± 106.45 | 381.04 ± 96.8 | 391.34 ± 109.67 | 378.26 ± 98.81 | 0.829 |
| Creatinine (μmol/L) | 59.59 ± 6.87 | 56.8 ± 9.05 | 59.52 ± 8.75 | 59.96 ± 9.18 | 0.648 |
| BUN (mmol/L) | 4.64 (3.76, 5.63) | 4.13 (3.28, 4.84) | 4.5 (3.84, 5.62) | 3.84 (3.49, 4.62) | 0.297 |
| Testosterone (nmol/L) | 1.92 (1.57, 2.65) | 2.23 (1.97, 2.89) | 2.11 (1.72, 2.5) | 2.03 (1.66, 2.51) | 0.574 |
| FSH (mIU/L) | 4.15 (3.46, 4.59) | 3.81 (3.26, 4.55) | 4.03 (3.5, 5.05) | 4.26 (3.44, 4.85) | 0.834 |
| LH (mIU/L) | 8.93 (5.15, 11.33) | 8.92 (5.53, 11.06) | 8.17 (4.56, 10.32) | 8.12 (6.16, 9.79) | 0.919 |
| Estradiol (pg/mL) | 52.5 (28.75, 70.5) | 55 (47.5, 90.5) | 42 (30, 61.5) | 50 (30.5, 70.5) | 0.307 |
| Progesterone (ng/mL) | 0.4 (0.3, 0.67) | 0.3 (0.2, 0.7) | 0.4 (0.2, 0.55) | 0.4 (0.3, 0.58) | 0.859 |
| PRL (ng/mL) | 19.48 (15.03, 24.49) | 13.35 (9.46, 24.64) | 17.4 (9.54, 19.62) | 12.32 (9.39, 19.37) | 0.448 |
| LH/FSH | 2.07 (1.4, 2.54) | 2.01 (1.73, 2.63) | 1.86 (1.27, 2.38) | 2.04 (1.65, 2.53) | 0.675 |
| SHBG (nmol/L) | 37.7 (16.98, 46.42) | 26 (22.83, 34.93) | 35.15 (17.45, 66.85) | 22.2 (11.9, 47.8) | 0.723 |
| DHEA-S (μg/dl) | 397.06 ± 139.96 | 424.59 ± 182.51 | 334.69 ± 201.79 | 363.42 ± 229.01 | 0.524 |
| FAI | 6.57 (3.1, 13.44) | 8.79 (4.95, 12.82) | 6.59 (3.92, 12.07) | 9.01 (4.71, 18.57) | 0.837 |
| CRP (mg/L) | 4.55 (1.78, 5) | 4.1 (2.62, 6.88) | 4.5 (3.6, 5) | 5 (4.25, 8.25) | 0.157 |
| IL-6 (pg/mL) | 4.4 (3.2, 8.32) | 4.7 (3.52, 8.38) | 3.8 (2, 5.62) | 3.7 (2.3, 6.88) | 0.616 |
| TNF-α (pg/mL) | 7.75 (5.52, 9.9) | 8.1 (7.4, 9.6) | 8 (5.97, 9.03) | 8.15 (7.42, 12.6) | 0.348 |
| IL-8 (pg/mL) | 7.03 (5, 27.8) | 6.1 (5.06, 10.27) | 12.2 (8, 17.3) | 10.72 (6.89, 39.75) | 0.186 |
| WBC (*10^9/L) | 6.39 ± 1.81 | 7.24 ± 1.58 | 6.44 ± 1.32 | 6.91 ± 1.89 | 0.342 |
| DIAPH1 (ng/mL) | 2.37 (2.34, 2.4) | 2.6 (2.51, 2.64) | 3.04 (2.86, 3.17)^a, c^ | 3.84 (3.5, 4.12)^b, d, e^ | < 0.001 |

Data are shown as mean ± SD for variables of normal distribution, and median with the interquartile range (25-75%) for skewed variables. For varibles of normal distribution, one-way ANOVA followed by Tukey multiple comparison test were performed considering four subgroups. For variables of skewed distribution, Kruskal-Wallis test followed by Pairwise comparisons using BWS All-Pairs Test were performed considering four subgroups. a: *P* < 0.05 for Q1 *vs* Q3. b: *P* < 0.05 for Q1 *vs* Q4. c: *P* < 0.05 for Q2 *vs* Q3. d: *P* < 0.05 for Q2 *vs* Q4. e: *P* < 0.05 for Q3 vs Q4.

Supplementary Table 6 Construction of regression models containing vital clinical indicators and DIAPH1.

|  | Model | Cut-off point | AUC | Sensitivity | Specificity | OR (95% CI) | *P* value |
| --- | --- | --- | --- | --- | --- | --- | --- |
| Model A | BMI+HOMA-β+Testosterone+DIAPH1 | 0.668 | 0.908 | 0.813 | 0.926 | 0.28 [0.11, 0.63] | 0.004 |
| Model B | BMI+FBG+LH/FSH+DIAPH1 | 0.514 | 0.881 | 0.827 | 0.853 | 0.32 [0.13, 0.69] | 0.006 |
| Model C | BMI+FBG+Testosterone+DIAPH1 | 0.368 | 0.913 | 0.920 | 0.809 | 0.21 [0.08, 0.49] | 0.001 |

AUC, area under the curve; OR, odd ratio; CI, confidence interval.

Supplementary table 7. The test method and equipment of clinical indicators.

| Test method | Equipment | Clinical indicators |
| --- | --- | --- |
| Hexokinase method | Automatic biochemical analyzer, Beckmann Kurt Co. LTD, USA | FBG |
| anion-exchange high-performance liquid chromatography. | Automatic hemoglobin A1c analyzer, Beckmann Kurt Co. LTD, USA | HbA1c |
| Chemiluminescence immunoassay | Automatic chemiluminescence immunoanalyzer, China Abbott Laboratories | Insulin, Testosterone, LH, FSH, DHEA-S, SHBG, Estradiol, Progesterone, PRL |
| Colorimetric method | Automatic biochemical analyzer, Beckmann Kurt Co. LTD, USA | TG, TC, HDL-C, LDL-C, ALT, AST, γ-GGT, UA, BUN, CREA |
| Fluorescent staining | Automatic modular blood and body fluid analyzer, Sysmex, Japan | WBC |
| IMMULITE 1000 Immunoassay System | Chemiluminescence immunoanalyzer, Siemens Healthcare Diagnostics Inc | CRP, IL-6, TNF-α, IL-8 |
